# Screening of Polyethylene-Degrading Bacteria from Rhyzopertha Dominica and Evaluation of Its Key Enzymes Degrading Polyethylene

**DOI:** 10.3390/polym14235127

**Published:** 2022-11-25

**Authors:** Yao Zhang, Yuan Lin, Hongmei Gou, Xu Feng, Xian Zhang, Lijuan Yang

**Affiliations:** 1 College of Bioengineering, Sichuan University of Science & Engineering, Yinbin 644000, China; 2 Liquor Making Bio-Technology & Application of Key Laboratory of Sichuan Province, Sichuan University of Science & Engineering, Yibin 644000, China

**Keywords:** polyethylene, *Acinetobacter baumannii* Rd-H2, laccase-like multi-copper oxidase, enzymatic properties, polyethylene degradation characterization

## Abstract

Polyethylene (PE) is widely used, and it has caused serious environmental problems due to its difficult degradation. At present, the mechanism of PE degradation by microorganisms is not clear, and the related enzymes of PE degradation need to be further explored. In this study, *Acinetobacter baumannii* Rd-H2 was obtained from Rhizopertha dominica, which had certain degradation effect on PE plastic. The degradation performance of the strains was evaluated by weight loss rate, SEM, ATR/FTIR, WCA, and GPC. The multi-copper oxidase gene abMco, which may be one of the key genes for PE degradation, was analyzed and successfully expressed in *E. coli*. The laccase activity of the gene was determined, and the enzyme activity was up to 159.82 U/L. The optimum temperature and pH of the enzyme are 45 °C and 4.5 respectively. It shows good stability at 30–45 °C. Cu^2+^ can activate the enzyme. The abMCO was used to degrade polyethylene film, showing a good degradation effect, proving that the enzyme could be the key to degrading PE.

## 1. Introduction

Plastic products are important consumer goods in daily life. Since 1950, the world’s production of non-fiber plastic products has reached 7300 tons, of which Polyethylene (PE) accounts for 36% [1]. Polyethylene is a large molecular weight polymer linked by a carbon–carbon single bond. It is chemically inert, corrosion resistant, non-toxic, recyclable and reprocessable [2]. High-density, low-density and linear low-density polyethylene is used in a wide range of life applications such as toys, bottles, water tanks, shopping bags, construction tools and disposable cups, as well as consumer products in electronics and medical fields [3]. All kinds of abandoned plastic products are exposed to the natural environment, forming plastic particles of different sizes which seriously pollute water resources and pose a serious threat to aquatic and Marine life [4]. The existing disposal methods of PE waste are mainly landfill and incineration [5]. As of 2015, about 6.3 billion tons of plastic waste had been generated worldwide, of which about 9 % was recycled, 12% was incinerated and 79% accumulated in landfills or the natural environment [6]. Landfill is simple but easy to occupy land and pollute groundwater. Animals may also swallow it as food, causing a variety of digestive tract diseases of livestock, and even more serious cases may lead to death. Incineration not only produces a large amount of air pollution but also can cause hazardous substances called dioxins, which can be slowly broken down into degradable substances for at least a year after entering the soil [7,8,9,10]. Therefore, it is urgent for us to find a method with high efficiency and no pollution to degrade PE plastics, which is difficult to degrade at present.

In recent years, researchers have isolated different strains from soil, ocean, and insect gut for plastic degradation. It has been reported that some bacteria such as *Pseudomonas* sp., *Bacillus* sp. and *Enterobacter Asburiae* et al. [11,12], *actinomycetes Nocardia* sp. et al. [13] and fungi such as *Fusarium Solani*, *Penicillium citrinum* PT1 have the potential to degrade PE. The main principle of microbial degradation of PE is that microorganisms have a powerful enzyme system and complex metabolic pathways, which are able to secrete extracellular enzymes that degrade complex structures, such as manganese peroxidase, laccase, alkane hydroxylase, alkane monooxygenase, ligninolytic enzyme, multi-copper oxidase, and finally catabolize plastic into CO_2_ and H_2_O [14,15]. Hirai et al. found that the laccase mediator system (LMS) using 1-hydroxybenzotriazole (HBT) as a mediator could significantly degrade high molecular weight polyethylene and nylon 66 membranes, so laccase was considered as the key enzyme in the degradation process of PE [16]. Santo proved that the breakage of polyethylene macromolecules could be catalyzed by enzymes secreted by microorganisms, and pointed out that laccase could act on polyethylene molecules to produce carbonyl groups, thereby reducing the molecular weight of polyethylene molecules [17]. H.V. Sowmay et al. found that laccase and manganese peroxidase were related to the degradation of polyethylene in the research of *Trichoderma harzianum* [18].

Laccase (EC 1.10.3.2) is a polyphenol oxidase with multiple copper ions in its catalytic center and belongs to the class of polycopper oxidases which is widely found in plants, insects, bacteria and fungi [19,20]. It can oxidize a series of substances including phenols, polyphenols, anilines, lignins, polycyclic aromatic hydrocarbons and even inorganic substances. Therefore, it is widely used in biobleaching, food industry, textile dye decolorization and plastic degradation industries [21]. Santo et al. found that Cu^2+^ induction could increase the biodegradation effect of laccase from Rhodococcus ruber on polyethylene [17]. Zhang et al. found that laccase derived from Antarctic chillophilic had a good effect on polyethylene, with a degradation rate of up to 13.2%, and the same Cu^2+^ could improve the activity of the enzyme 127.10% [22].

Rhizopertha dominica is a dangerous pest of storage grains and foods. It was found that Rhizopertha dominica can destroy PE membrane by chewing and phagocytosis. Given that PE degrading strains have been obtained from the intestinal tract of insects (waxworms or Indian mealmoth), it is speculated that there are also microorganisms capable of digesting PE in Rhizopertha dominica. It is proposed to screen the microorganisms that can effectively degrade PE from Rhizopertha dominica and explore the degradation of PE by enzymes secreted by microorganisms, in order to enrich the microbial resources of degraded PE, excavate new laccase, and provide a reference for the mechanism of microbial degradation of PE.

## 2. Materials and Methods

### 2.1. PE Material and Culture Medium

The PE film is a conventional food fresh-keeping bag film, with a weight-average molecular weight (Mw) greater than 130,000, belonging to low-density polyethylene (LDPE), and the weight-average molecular weight (Mw) of PE powder is greater than 80,000. The PE films were cut into 4.0 cm × 4.0 cm, 1.0 cm × 1.0 cm pieces. They were soaked in 0.5% potassium chloride for 1 h. The remaining potassium chloride was rinsed off with ethanol and then rinsed with sterile water. The PE films were dried at 50 °C, and finally the PE films were put under a UV lamp for 4 h for sterilization. Liquid carbon-free basal medium (LCFBM) and Luria–Bertani (LB) medium was prepared with deionized water. Each 1000 mL of LCFBM contains K_2_HPO_4_ 0.7 g, KH_2_PO_4_ 0.7 g, MgSO_4_ 7H_2_O 0.7 g, NH_4_NO_3_ 1.0 g, FeSO_4_ 7H_2_O 0.002 g, ZnSO_4_ 7H_2_O 0.002 g, MnSO_4_ H_2_O 0.001 g, NaCl 0.005 g. Carbon-free basal agar medium (CFBAM) was prepared by adding 20 g of agar to 1000 mL of LCFBM. Prepared cultures were autoclaved at 121 °C for 20 min.

### 2.2. Preliminary Screening of PE-Degrading Strains

The surface of Rhizopertha dominica was sterilized with medical ethanol and then washed three times with 0.9% NaCl. Next, the Rhizopertha dominica was ground and crushed into a centrifuge tube containing 10 mL of 0.9% NaCl. After shaking on a vortex oscillator for 5 min, the worm tissues were removed by filtration. Then, 5 mL of the upper liquid was taken into LB medium (50 mL/250 mL conical flask), and incubated at 30 °C, 180 r/min in a constant temperature shaker for 1 d to complete the enrichment of microorganisms. CFBAM (containing 10 g/L PE powder) was prepared, and the enriched bacterial solution was diluted and spread on CFBAM plates containing PE powder and incubated at 30 °C for 3 d. After the strains had grown, single colonies were picked out and scribed onto plates of CFBAM containing PE powder. The steps of the above operation were repeated 3 times. The better growing single strains were isolated and selected, and the colony morphology was compared in size and color under the microscope. The different morphological strains were numbered and stored at 4 °C and numbered.

Accurately weigh the initial weight of the prepared PE film and number it, add it to LCFBM (50 mL/250 mL conical flask) after sterilization, activate the screened strain, and inoculate it into the PE film at an inoculum of 5%. In the LCFBM of 30 °C, 160 r/min constant temperature shaking culture for 30 d, each strain was made three parallels. The PE film was taken out from the 30-day co-culture solution, soaked in 2% SDS solution for 4 h, and repeatedly washed with sterile water for several times to remove the microorganisms attached to the surface of the film. After drying the surface moisture in an oven at 40 °C, take it out and weigh it in time. According to the weighing results, the weight loss rate of the PE film was calculated, and the weight loss rate = (m_1_ − m_2_)/m_1_, where m_1_ was the initial weight of the PE film, and m_2_ was the weight of the PE film after 30 days. The strain with the highest weight loss rate was selected for subsequent determination.

### 2.3. Identification of PE Degrading Strains

The selected PE-degrading strains were seeded in LB medium at 37 °C and 180 r/min for 12 h, and the colony morphology and Gram staining were observed, and the bacteria were collected using the bacterial DNA genome extraction kit to extract the genome of the strain, then PCR amplification of 16S rRNA was carried out. The universal primers were 27-F (5′-CAGAGTTTGATCCTGGCT-3′) and 1492-R (5′-AGGAGGTGATCCAGCCGCA-3′). The PCR products were detected by 1% agarose gel electrophoresis and sent to Chengdu Qingke Company for sequencing. Sequencing results were compared with microorganisms in the GenBank database using the Basic Local Alignment Search Tool (BLAST) created by the National Center for Biotechnology Information (NCBI). The reliable strain sequences with high similarity were selected, and the phylogenetic tree was constructed by the neighbor-joining method of MEGA 7 software.

### 2.4. Determination of PE Degradation Properties

Take out the PE film with the highest weight loss rate measured in 2.2. The surface morphology was observed by SEM, and the change of chemical functional groups on the surface of the PE film was determined by ATR/FTIR. In addition, a change in the polarity of the PE film was found in the WCA experiment. The changes in molecular weight before and after the degradation of the PE powder were determined using GPC. The PE films or PE powders that were not inoculated with the degrading strains and cultured in LCFBM for 30 days were used as controls.

### 2.5. Heterologous Expression of the Laccase-like Multicopper Oxidase

The AbMco gene was amplified by PCR using *Acinetobacter baumannii* Rd-H2 genomic DNA as a template, and the upstream and downstream primers were AbMco-F/AbMco-R (AbMco-F: 5′-CGCGGATCCGCAATTAAGGAATATCACCTTA-3′; AbMco-R:5′-CCCAAGCTTTTAGTGATGCGCATGACTAGCTC-3′). The PCR product was purified and digested with *BamH* Ⅰ and *Hind* III, and then connected to the expression plasmid pET-28a to construct a recombinant expression plasmid pET-28a-AbMco, which was transformed into *E. coli* DH5α, and the recombinant was identified by double digestion, the positive recombinant plasmids were sent to Qingke Biological Company for sequencing. The recombinant expression plasmid pET-28a-AbMco was transformed into *E. coli* BL21 (DE3), and the recombinant *E. coli* BL21/pET-28a-AbMco was cultured overnight and activated. The seed liquid of the cultured recombinants was inoculated into a fresh LB medium (containing 50 μg/mL kanamycin). The cells were incubated at 37 °C with shaking at 180 rpm for 2–3 h, added with 1 mM isopropyl-β-D-thiogalactoside (IPTG), and incubated for 8 h at 25 °C and 180 rpm. The recombinant *E. coli* BL21/pET-28a-AbMco was collected at 4 °C and 7000 rpm. The cells were washed twice with 20 mM PBS (pH 7.4), resuspended, and sonicated. The disrupted bacterial solution was centrifuged at 4 °C and 7000 rpm for 30 min, and the crude enzyme solution of AbMCO was collected.

### 2.6. Multi-Copper Oxidase Laccase Activity Assay

The enzyme activity of AbMCO was determined by the visible light absorption method: 2,2′-azo-bis-3-ethyl benzothiazoline 6-sulfonic acid (ABTS) was used as the substrate to measure the change of *OD* value within 3 min at 420 nm. The reaction system consisted of 0.2 mL enzyme solution, 3.8 mL buffer containing 0.5 mmol/L ABTS, and 1 mmol/L HAK-NAAC. The amount of enzyme required to oxidize 1 μmol of ABTS per minute was defined as one unit of enzyme activity (U) [23]. The calculation formula of enzyme activity is as follows:

$$\mathrm{Enzyme}\;\mathrm{activity}\;\left(U/L\right)=10^{6}\times \frac{V_{0}\times \Delta OD\times n}{\Delta t\times V_{1}\times \varepsilon }$$

In the formula: Δ*OD* is the change value of *OD*420; Δ*t* is the reaction time (min); *V*_1_ is the amount of enzyme (0.2 mL); *V*_0_ is the total volume of the reaction system (4 mL); *n* is the dilution ratio of the enzyme solution; ε is the Molar extinction coefficient (*ε* = 3.6 × 10^4^ L·mol^−1^·cm^−1^).

### 2.7. Effects of Temperature and pH on the Activity and Stability of Multicopper Oxidase

The enzyme activity of AbMCO was measured at different temperatures (30–60 °C) to determine the optimal reaction temperature of the enzyme. The enzyme activity was measured after the enzyme solution was incubated at different temperatures for 0.5 h, and the relative enzyme activity of the enzyme was calculated by taking the initial enzyme activity of the enzyme solution at each temperature as 100% to determine the effect of temperature on the stability of the enzyme. The HAc-NaAc buffer solution (containing 0.5 mmol/L ABTS) with pH of 3.0–6.0 was prepared, and the enzyme activity of the enzyme solution under various pH conditions was measured at 37 °C to determine the optimal pH of the enzyme. Taking the initial enzyme activity of the enzyme at each pH as 100%, the effect of pH on the enzyme stability was determined.

### 2.8. Effect of Metal Ions on Enzyme Activity

The enzyme solution was mixed with 1 mmol/L metal ion (K^+,^ Cu^2+^, Ca^2+^, Fe^2+^, Mg^2+^, and Mn^2+^) buffer solution, and the enzyme activity was measured after incubating at 37 °C for 10 min.

### 2.9. Characterization of PE Degradation by Laccase-like Multi-Copper Oxidase

The PE film with a size of 1.0 cm * 1.0 cm treated in 2.1 was mixed with the AbMCO initial enzyme solution, placed in a 42 °C water bath for 12 h, washed repeatedly with alcohol and sterile water after the water bath, and then soaked in 2% SDS. After 4 h, it was repeatedly washed with alcohol and sterile water to remove surface-attached proteins, and dried in an electric heating blast drying oven at 42 °C. The treated PE films were measured by scanning electron microscope (SEM), surface attenuated total reflection infrared spectroscopy (ATR/FTIR), water contact angle (WCA), and high-temperature gel permeation chromatography (GPC).

## 3. Results and Discussion

### 3.1. Screening and Identification of PE-Degrading Strains

**Figure 1:**
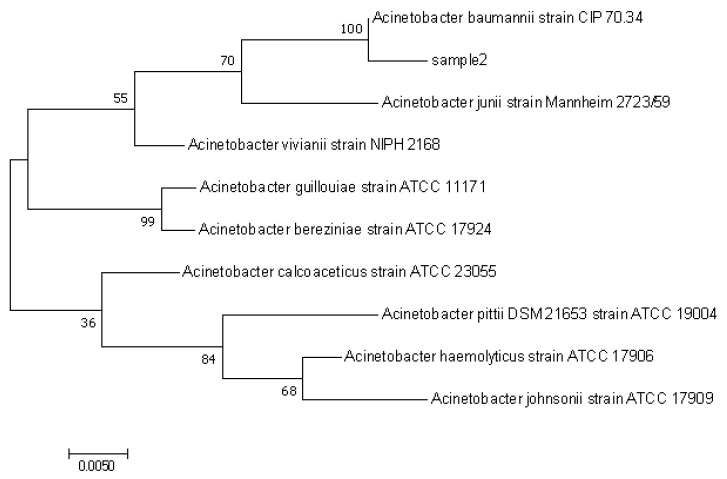
Neighbor-Joining method was used to infer the evolutionary history of strains sample2 (Rd-H2).

In the medium with PE as the only carbon source, eight strains of bacteria were isolated from Rhyzopertha dominica, and these eight strains were numbered Rd-H1 to Rd-H8. After repeated purification and taking the weight loss rate of PE film for 30 days as the re-screening condition, it was finally found that strain Rd-H2 had the best degradation effect on PE, and the weight loss rate of PE was 0.62 ± 0.062% (Table 1). After treating culture twice on CFBAM and treating separation and purification three times on LB AGAR, the morphology of the Rd-H2 colony was gray, smooth and round with neat edges. The Rd-H2 strain was Gram-stained, red, and was judged as Gram-negative bacteria (Figure S1). The genome of Rd-H2 was extracted, and 16S rRNA was amplified by PCR and sequenced. The length of PCR amplified fragment was about 1400 bps. The PCR products were purified and sent to Chengdu Qing Ke Company for sequencing. The sequencing results were compared by BLAST on NCBI, and the homologous gene sequences with more than 95% similarity were selected. The “N-J neighborhood method” in MEGA 7 was used to construct the phylogenetic tree. Strain Rd-H2 and model strain *Acinetobacter baumannii* strain CIP 70.34 were in the same phylogenetic branch, with a self-developing value of 100 and high confidence (Figure 1). It was concluded that strain MK-1 was closely related to *Acinetobacter baumannii*. Strain Rd-H2 was identified as *Acinetobacter baumannii* by analyzing the size, morphology, Gram staining and molecular identification results of the strain, so it was named as *Acinetobacter baumannii* Rd-H2 (Rd-H2).

### 3.2. Degradation Performance of Rd-H2 on LDPE Films

**Figure 2:**
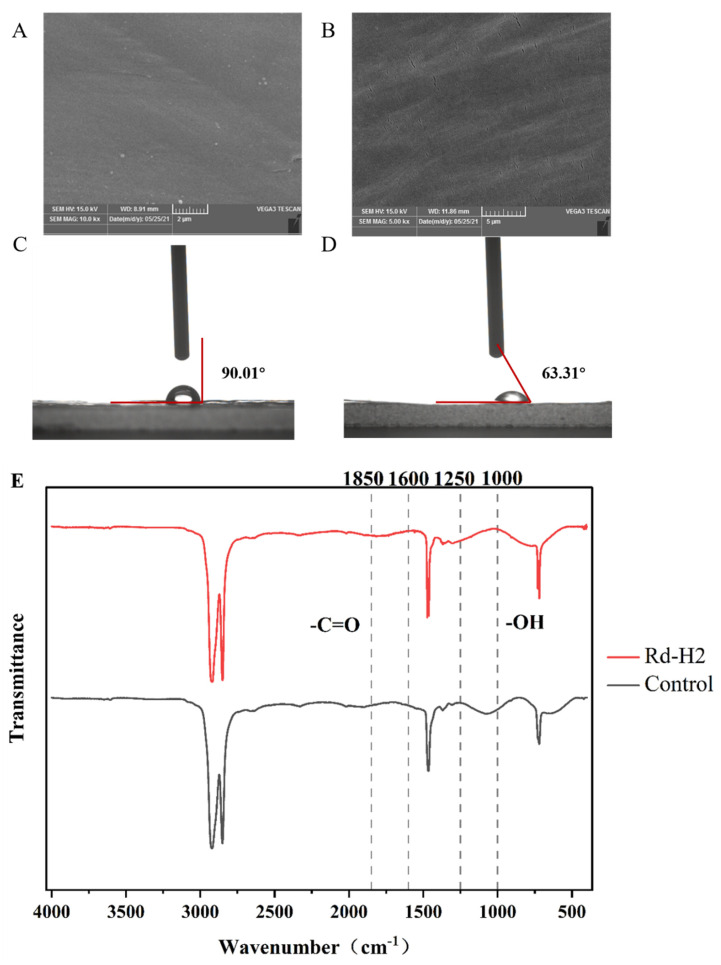
Characterization of PE film degradation by strain Rd-H2. (A and B) SEM observations of the physical surface topography of the sterile control (**A**) versus the PE sheets incubated with strains Rd-H2 (**B**) after 30 days. (**C**,**D**) WCA observation of physical surface hydrophilicity of the sterile control (**C**) versus the PE sheets incubated with strains Rd-H2 (**D**) after 30 days. (**E**) ATR-FTIR spectra analysis of the sterile control and PE samples after the 30-day incubation with strains Rd-H2.

After 30 days of incubation, the biofilm was completely removed from the PE samples, and the changes in the surface morphology of the PE film were observed by SEM, The surface of the PE film in the control group without the inoculated strain was smooth and complete without obvious changes. The surface of the PE film inoculated with the strain Rd-H2 became rough and uneven, with cracks and creases (Figure 2A,B). Similar to previous studies using bacteria to degrade LDPE [24], this result indicates that this strain does damage the physical integrity of PE films [25,26].

WCA can assist to prove the degradation of PE by microbial strains. There is an inverse relationship between the size of the contact Angle and hydrophilicity. The smaller the contact angle, the more favorable it is for microorganisms to attach to the surface of PE film. After 30 days of co-culture with PE degrading bacteria, the contact Angle θ of the control group was 90°, and that of the experimental group was 60.31° (Figure 2C,D), and the contact angle decreased significantly. The decrease of hydrophobicity of PE film will reduce the resistance to bacterial degradation, making it more conducive to the attachment and fixing of PE, so as to improve the degradation rate of PE [27,28].

FTIR was used to detect the changes of functional groups or surface chemical structures of PE films. After co-culture and incubation of PE films with strain Rd-H2 for 30 days, in the range of 1000–1250 cm^−1^, a comparison was made with the control, which shows that the reduction of C-O stretching due to the alcohol (-OH) group leads to the disappearance of the signal peak in the sample group [29]. The characteristic peak of the carbonyl group appeared on the surface of PE film, and the carbonyl group range (1600 cm^−1^~1850 cm^−1^) decreased exponentially (Figure 2E). The research shows that the appearance of carbonyl group is an important sign of PE biodegradation [30]. The carbonyl group is formed by the presence of oxygen atoms in the carbon chain of the polymer. The reduction of the original chemical bonds and the formation of new bonds indicates that the structure of PE has changed, the structure has become loose, and its energy level has been reduced. The research shows that the appearance of carbonyl group is an important sign of PE biodegradation. Based on these key chemical bond changes, we conclude that the primary structural changes in the Rd-H2-treated PE membranes occurred, representing direct biodegradation by bacteria. Bacteria treatment causes PE polymer chain breakage, which reduces the molecular weight and increases the hydrophilicity of th e PE polymer.

According to the GPC results (Table 2), strain Rd-H2 reduced the Mn of PE powder from 19,979 to 18,158, the Mw from 81,315 to 79,244, the PD from 4.04 to 4.37, and the Mw of PE powder decreased by 2.54%. The molecular weight of PE powder in the GPC results was decreasing, and the increase of diversity dispersion index proved that the PE powder was depolymerized by the treatment of the strain, and the strain had some degradation effect on PE polymer.

### 3.3. Cloning and Expression of AbMco Gene

**Figure 3:**
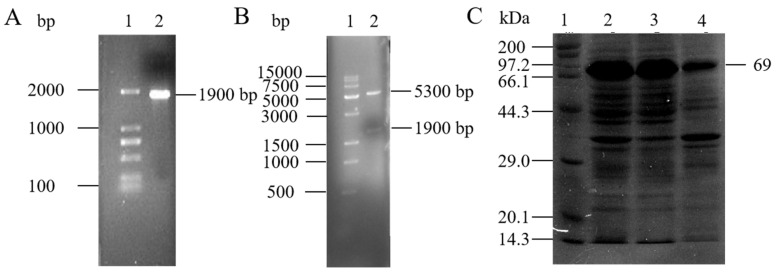
Cloning and expression of multicopper oxidase (abMco) from *Acinetobacter baumannii* Rd-H2. (**A**) PCR amplification results of abMCO. Lane 1: DNA marker (100–2000 bps); Lane 2: abMco amplification results (1900 bps). (**B**) The recombinant plasmid pET-28a-abMCO was digested with *BamH* I and *Hind* III restriction enzymes. Lane 1: DNA marker (500–15,000 bps); Lane 2: Double digestion of the pET-28a-abMco sequence with an insertion size of 1900 bps. (**C**) Analysis of multiple copper oxidase (abMCO) by SDS-PAGE. Lane 1: Standard protein marker; Lane 2: IPTG induced crude protein expressed by E. coli carrying plasmid pET-28a-abMCO; Lane 3: IPTG-induced ultrasound-treated supernatant of *E. coli* carrying plasmid pET-28a-abMCO; Lane 4: IPTG-induced ultrasound-treated precipitation of *E. coli* carrying plasmid pET-28a-abMCO.

Laccase is a copper-containing polyphenol oxidase, and it is the most important member of the multi-copper oxidase family. Numerous studies have shown that it plays an important role in the biodegradation of PE [16,17,31]. The study searched and analyzed the NCBI database for the polycopper oxidase gene (GeneBanK ID: WP_000018555.1) of *Acinetobacter baumannii* based on the available reports on the application of polycopper oxidase for plastic degradation. The polycopper oxidase protein from A. baumannii consists of 644 amino acid residues without a signal peptide and has a theoretical isoelectric point (pI) and subunit Mm of 6.59 and 72.6 kDa, respectively, on the ExPASy website (https://web.expasyorg/protparam/, accessed on 13th November 2022). The multiple copper oxidase gene from *Acinetobacter baumannii* Rd-H2 was speculated, and the performance of the enzyme on the degradation of PE plastic was verified. The genomic DNA of Rd-H2 was obtained and amplified by PCR with specific primers, and a single strand of about 1.9 kbps was obtained (Figure 3A). The PCR product was cloned into the expression plasmid pET-28a and transferred into *E. coli* DH5α competent cells. The positive transformants were selected, and the recombinant expression plasmid pET-28a-abMco was extracted for restriction enzyme digestion. Two bands of about 5300 bps and 1900 bps appeared in the product after restriction enzyme digestion with *BamH* I and *Hind* III, and the experimental values were in accordance with the theoretical values, indicating that the vector pET28a-abMco was successfully constructed (Figure 3B). The above clones were induced by 1 mM IPTG, and the molecular weight of Polychlorinated Oxidase determined by SDS-PAGE was 69 kDa, which was consistent with the theoretical value (Figure 3C). At 37 °C, the laccase activity of the supernatant was 159.82 U/L. 

### 3.4. Effects of Temperature and pH on the Enzymatic Activity and Stability of Multicopper Oxidase

**Figure 4:**
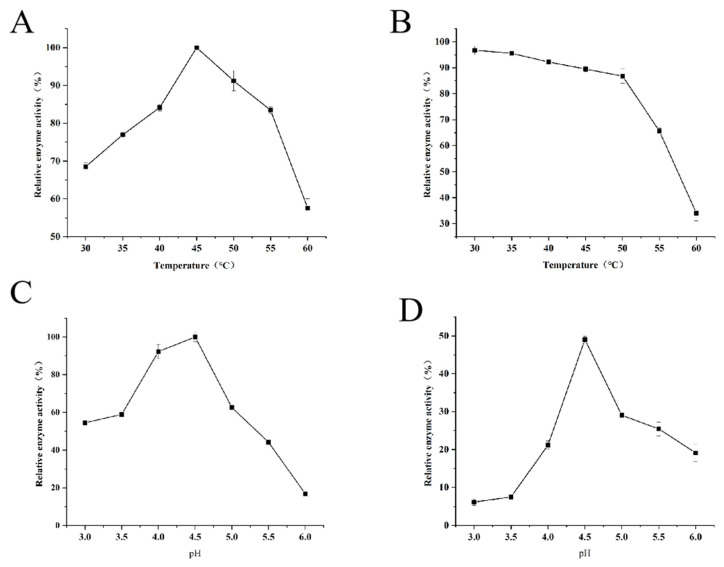
Determination of enzymatic properties of multicopper oxidase (abMCO). (**A**) Effect of different temperatures on enzymatic activity of abMCO (**B**) The temperature stability of abMCO. (**C**) Effect of different pH on enzymatic activity of abMCO. (**D**) The pH stability of abMCO.

The enzyme activity of abMCO was measured at the temperature of 30–60 °C (Figure 4A). It was found that the optimum temperature of abMCO was 45 °C, and when the temperature was higher than 50 °C, the enzyme activity decreased sharply. The reported optimum temperature of laccase varies greatly depending on species [23,32,33]. The stability of laccase at 30–60 °C is shown in Figure 4B. After the enzyme is kept at 30–40 °C for 0.5 h, the residual activity of laccase is more than 85%. When the temperature is higher than 50 °C, the stability of the enzyme begins to decline. The enzyme showed higher activity at lower pH (Figure 4C). The enzyme showed the highest enzyme activity at pH 4.5. The optimum pH of enzymes from different sources also showed species differences [23,34]. The pH stability of the enzyme is an important index in the practical application of the enzyme. After the enzyme is stored at 4 °C for 4 h under different pH conditions, the residual activity of the enzyme is measured at pH 4.5 (Figure 4D). The residual activity of this enzyme is about 50% at pH 4.5. When the pH value is greater than 5.0, the stability of the enzyme decreases rapidly, and the residual activity of the enzyme only accounts for 18% of the initial value at pH 6.

### 3.5. The Effect of Metal Ions on the Activity of Multicopper Oxidase

In this study, the effect of metal cations on the activity of abMCO was investigated (Table 3). Cu^2+^ can activate abMCO. The multi-copper oxidase can bind to copper ions and then use the unique redox ability of copper ions to react with the substrate in a redox reaction, thus increasing the enzyme activity [19]. The rest of Mn^2+^, K^+^, Mg^2+^ and Fe^2+^ have inhibitory effects on the activity of abMco, among which Mg^2+^ has the most significant inhibitory effect. The metal ions may complex with N, O atoms, etc. on the protein to generate complexes that cause the peptide chain to bend to ensure that the coordination atoms are in the proper position. Because the metal ion coordination leads to structural changes, it is unable to perform its function properly, thus leading to degradation of enzyme activity. As with the previous results, the presence of Cu^2+^ can improve the activity of laccase. Zhang et al. found that laccase from *Bacillus vallismortis* fmb-103 and Javadzadeh et al. from *Bacillus* sp. CF96 can promote laccase activity in the presence of Mn^2+^ and Cu^2+^. K^+^ can inhibit laccase activity [35,36]. Different from the results, it is reported that the presence of Mn^2+^ and Mg^2+^ can increase the activity of laccase, but the presence of Mn^2+^ and Mg^2+^ in this study inhibits the activity of laccase.

### 3.6. Degradation Performance of Multi-Copper Oxidase on PE Film

**Figure 5:**
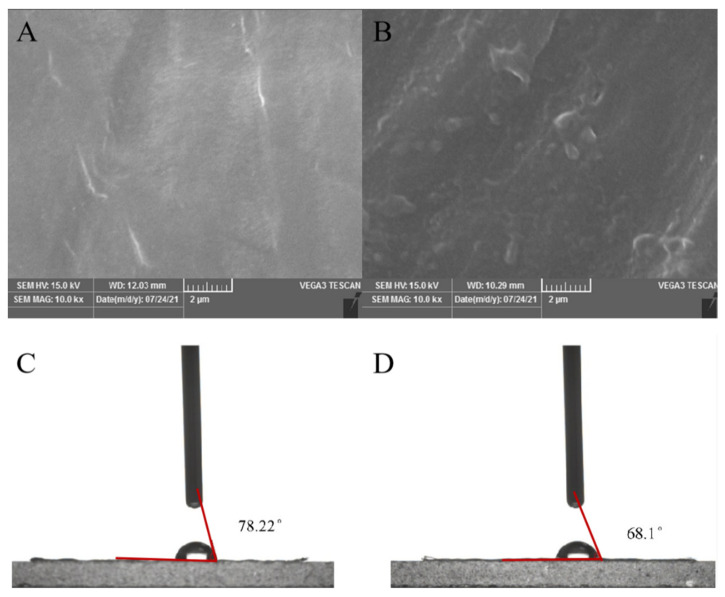
Characterization of PE degradation by multiple copper oxidase (abMco). (**A**,**B**) SEM results; (**A**) PE film without abMco treatment; (**B**) PE film treated with abMco. (**C**,**D**) WCA results; (**C**) PE film without abMco treatment, with a contact Angle of 78.22°; (**D**) The contact Angle of PE film treated with abMco is 68.1°.

Taking the PE film treated with pET-28a empty plasmid expression supernatant as the control group, after being treated with abMCO crude enzyme solution (Figure 5A,B), the surface of PE film in the experimental group showed obvious wrinkles, roughness and holes, while the control group was still smooth. SEM results showed that abMCO could cause some damage to the surface morphology of PE film. In the test of the contact angle of the treated PE film mixed with the enzyme solution (Figure 5C,D), the contact angle θ of the comparative control group is 78.22° and that of the experimental group is 68.10°. In addition, in the results of GPC (Table 4), Mn (16,347), Mw (134,314) of the experimental group PE film decreased by 52.20% and Mw decreased by 1% compared with Mn (34,197) and Mw (135,592) of the control group PE film. The PD value increased from 3.97 to 8.22. The decrease of Mn and Mw values indicated that abMCO had a certain degradation effect on PE and compared with the GPC results of the previous strain for PE film degradation, the change of Mn was greater, which proved that the multi-copper oxidase of strain Mk-1 had a good degradation effect on PE film.

## 4. Conclusions

In this study, biodegradation of unprepared polyethylene material was carried out using newly isolated *Acinetobacter baumannii* and a multi-copper oxidase derived from this bacterium. The degradation of the polyethylene material by A. baumannii and ab MCO was confirmed by weight loss, SEM, WAC, FTIR and GPC techniques. In addition, the biochemical properties of abMCO were investigated and it was found that the enzyme has good stability at 30–45 °C and Cu^2+^ has an activating effect on the enzyme. Further studies on the complete degradation of polyethylene are needed to reveal the mechanism of degradation of polyethylene plastic by multi-copper oxidase. In conclusion, the results of this study provide new strain resources as well as enzyme resources for the study of microbial degradation of polyethylene.

## Figures and Tables

**Table 1:** Effect of metal ions on AbMCO enzyme activity.

Serial Number	Weight Loss Rate/%
Rd-H1	0.16 ± 0.08
Rd-H2	0.62 ± 0.062
Rd-H3	0.49 ± 0.020
Rd-H4	0.20 ± 0.12
Rd-H5	0.50 ± 0.002
Rd-H6	0.21 ± 0.014
Rd-H7	0.19 ± 0.012
Rd-H8	0.21 ± 0.012

**Table 2:** Analysis of GPC assay of PE powder after 30 days of Rd-H2 treatment.

Name	Control	Rd-H2 (30 d)
Mn	19,979	18,158
Mw	81,315	79,244
PD	4.04	4.37

**Table 3:** Effect of metal ions on abMCO enzyme activity.

Metal Ion (5 mmol/L)	Relative Activity/(%)
Control	100 ± 0.73
K^+^	88.26 ± 1.40
Mg^2+^	62.46 ± 0.55
Fe^2+^	92.63 ± 0.92
Mn^2+^	95.80 ± 0.057
Cu^2+^	102.14 ± 0.75

**Table 4:** Analysis of GPC detection of PE films after abMCO treatment.

Name	Control	abMCO
Mn	34,197	16,347
Mw	1,355,592	134,314
PD	3.97	8.22

## Data Availability

Date is contained within the article.

## Supplementary Materials

The following supporting information can be downloaded at: https://www.mdpi.com/article/10.3390/polym14235127/s1, Figure S1: Colony morphology of *Acinetobacter Baumannii* Rd-H2 in the medium(A) and Gram staining results of *Acinetobacter Baumannii* Rd-H2 under microscope(B); Figure S2: GPC analysis of the PE treated by the LCFBM medium incubated without (A) or with (B) strain Rd-H2 for 30 days. Figure S3: GPC analysis of the PE treated without (A) or with (B) AbMCO for 10h.

## References

[1] Wong Y., Yu J. (1999). Laccase-catalyzed decolorization of synthetic dyes. Water Res..

[2] Kong D., Wang L., Chen X., Xia W., Su L., Zuo F., Yan Z., Chen S., Wu J. (2022). Chemical-biological degradation of polyethylene combining Baeyer–Villiger oxidation and hydrolysis reaction of cutinase. Green Chem..

[3] Mahmood Q., Li X., Qin L., Wang L., Sun W.-H. (2022). Structural evolution of iminopyridine support for nickel/palladium catalysts in ethylene (oligo)polymerization. Dalton Trans..

[4] Moharir R.V., Kumar S. (2019). Challenges associated with plastic waste disposal and allied microbial routes for its effective degradation: A comprehensive review. J. Clean. Prod..

[5] Jiakang R., Yixin H., Yu Y. (2020). Microbial Degradation and Valorization of Plastic Wastes. Front. Microbiol..

[6] Geyer R., Jambeck J.R., Law K.L. (2017). Production, use, and fate of all plastics ever made. Sci. Adv..

[7] Alshehrei F. (2017). Biodegradation of Synthetic and Natural Plastic by Microorganisms. J. Appl. Environ. Microbiol..

[8] Royer S.J., Ferrón S., Wilson S.T., Karl D.M. (2018). Production of methane and ethylene from plastic in the environment. PLoS ONE.

[9] Webb H.K., Arnott J., Crawford R.J., Ivanova E.P. (2012). Plastic Degradation and Its Environmental Implications with Special Reference to Poly(ethylene terephthalate). Polymers.

[10] Chamas A., Moon H., Zheng J., Qiu Y., Tabassum T., Jang J.H., Abu-Omar M., Scott S.L., Suh S. (2020). Degradation Rates of Plastics in the Environment. ACS Sustain. Chem. Eng..

[11] Tribedi P., Sil A.K. (2013). Low-density polyethylene degradation by *Pseudomonas* sp. AKS2 biofilm. Environ. Sci. Pollut. Res..

[12] Yang J., Yang Y., Wu W.M., Zhao J., Jiang L. (2014). Evidence of Polyethylene Biodegradation by Bacterial Strains from the Guts of Plastic-Eating Waxworms. Environ. Sci. Technol..

[13] Liu Y., Yuan Z., Zhai J., Li R., Huo Y., Zhang X., Jing W. (2020). Screening, identification and degradation characteristics of polyethylene degrading bacteria in soil. Acta Microbiol. Sin..

[14] Zhang J., Gao D., Li Q., Zhao Y., Li L., Lin H., Bi Q., Zhao Y. (2020). Biodegradation of polyethylene microplastic particles by the fungus Aspergillus flavus from the guts of wax moth Galleria mellonella. Sci. Total Environ..

[15] Ameen F., Moslem M., Hadi S., Al-Sabri A.E. (2015). Biodegradation of Low Density Polyethylene (Ldpe) by Mangrove Fungi from the Red Sea Coast. Prog. Rubber Plast. Recycl. Technol..

[16] Fujisawa M., Hirai H., Nishida T. (2001). Degradation of Polyethylene and Nylon-66 by the Laccase-Mediator System. J. Polym. Environ..

[17] Santo M., Weitsman R., Sivan A. (2013). The role of the copper-binding enzyme–laccase–in the biodegradation of polyethylene by the actinomycete Rhodococcus ruber. Int. Biodeterior. Biodegrad..

[18] Sowmya H.V., Ramalingappa, Krishnappa M., Thippeswamy B. (2014). Degradation of polyethylene by Trichoderma harzianum—SEM, FTIR, and NMR analyses. Environ. Monit. Assess..

[19] Giardina P., Faraco V., Pezzella C., Piscitelli A., Vanhulle S., Sannia G. (2010). Laccases: A never-ending story. Cell. Mol. Life Sci..

[20] Claus H. (2003). Laccases and their occurrence in prokaryotes. Arch. Microbiol..

[21] Kai S., Shunyao L., Youbin S., Qingguo H. (2021). Advances in laccase-triggered anabolism for biotechnology applications. Crit. Rev. Biotechnol..

[22] Zhang A., Hou Y., Wang Q., Wang Y. (2022). Characteristics and polyethylene biodegradation function of a novel cold-adapted bacterial laccase from Antarctic sea ice psychrophile *Psychrobacter* sp. NJ228. J. Hazard. Mater..

[23] Bourbonnais R., Paice M.G. (1990). Oxidation of non-phenolic substrates. An expanded role for laccase in lignin biodegradation. FEBS Lett..

[24] Sridharan R., Krishnaswamy Veena G., Senthil K.P. (2021). Analysis and microbial degradation of Low-Density Polyethylene (LDPE) in Winogradsky column. Environ. Res..

[25] Ojha N., Pradhan N., Singh S., Barla A., Shrivastava A., Khatua P., Rai V., Bose S. (2017). Evaluation of HDPE and LDPE degradation by fungus, implemented by statistical optimization. Sci. Rep..

[26] Khandare S.D., Agrawal D., Mehru N., Chaudhary D.R. (2022). Marine bacterial based enzymatic degradation of low-density polyethylene (LDPE) plastic. J. Environ. Chem. Eng..

[27] Gilan I., Hadar Y., Sivan A. (2004). Colonization, biofilm formation and biodegradation of polyethylene by a strain of Rhodococcus ruber. Appl. Microbiol. Biotechnol..

[28] Lou H., Fu R., Long T., Fan B., Guo C., Li L., Zhang J., Zhang G. (2022). Biodegradation of polyethylene by Meyerozyma guilliermondii and Serratia marcescens isolated from the gut of waxworms (larvae of *Plodia interpunctella*). Sci. Total Environ..

[29] Copinet A., Bertrand C., Govindin S., Coma V., Couturier Y. (2004). Effects of ultraviolet light (315 nm), temperature and relative humidity on the degradation of polylactic acid plastic films. Chemosphere.

[30] Gao R., Sun C. (2021). A marine bacterial community capable of degrading poly(ethylene terephthalate) and polyethylene. J. Hazard. Mater..

[31] Sowmya H.V., Ramalingappa, Krishnappa M., Thippeswamy B. (2015). Degradation of polyethylene by Penicillium simplicissimum isolated from local dumpsite of Shivamogga district. Environ. Dev. Sustain..

[32] Wang H., Huang L., Li Y., Ma J., Wang S., Zhang Y., Ge X., Wang N., Lu. F., Liu Y. (2020). Characterization and application of a novel laccase derived from Bacillus amyloliquefaciens. Int. J. Biol. Macromol..

[33] Telke A.A., Ghodake G.S., Kalyani D.C., Dhanve R.S., Govindwar S.P. (2011). Biochemical characteristics of a textile dye degrading extracellular laccase from a *Bacillus* sp. ADR. Bioresour. Technol..

[34] Neelkant K.S., Shankar K., Jayalakshmi S.K., Sreeramulu K. (2020). Purification, biochemical characterization, and facile immobilization of laccase from Sphingobacterium ksn-11 and its application in transformation of diclofenac. Appl. Biochem. Biotechnol..

[35] Zhang C., Zhang S., Diao H., Zhao H., Zhu X., Lu F., Zhaoxin L. (2013). Purification and characterization of a temperature- and pH-stable laccase from the spores of Bacillus vallismortis fmb-103 and its application in the degradation of malachite green. J. Agric. Food Chem..

[36] Javadzadeh S.-G., Asoodeh A. (2020). A novel textile dye degrading extracellular laccase from symbiotic bacterium of *Bacillus* sp. CF96 isolated from gut termite (Anacanthotermes). Int. J. Biol. Macromol..

