# Supplementary material for: Screening of Polyethylene-Degrading Bacteria from Rhyzopertha Dominica and Evaluation of Its Key Enzymes Degrading Polyethylene
Source: Polymers (Basel). 2022 Nov 25;14(23):5127. doi: 10.3390/polym14235127 (PMC9736035; doi:10.3390/polym14235127)
Supplement: Supplementary file 1 [file polymers-14-05127-s001.zip › polymers-2029225-supplementary.pdf]

polymers

MDPI

Supplementary information

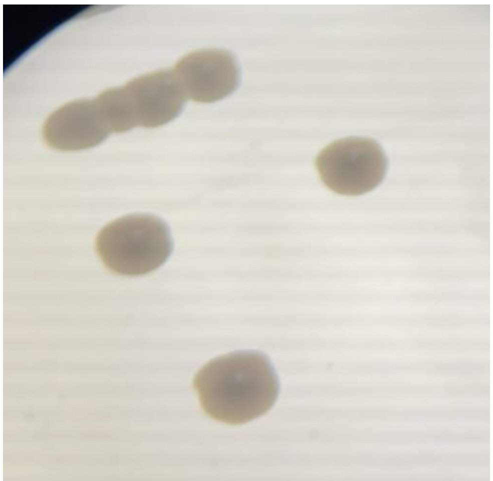

(A)

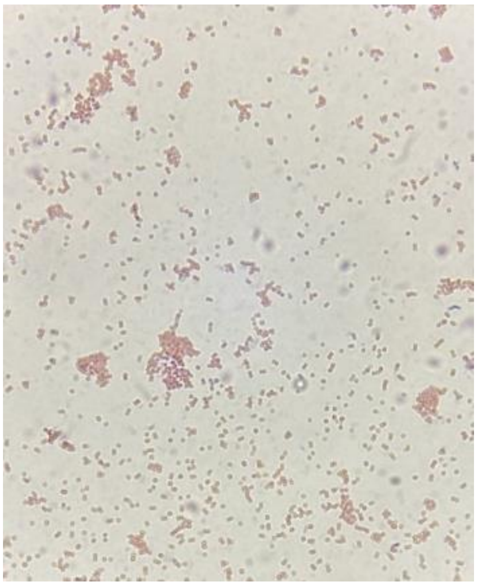

(B)

Figure S1. Colony morphology of *Acinetobacter Baumannii* Rd-H2 in the medium(A) and Gram staining results of *Acinetobacter Baumannii* Rd-H2 under microscope(B).

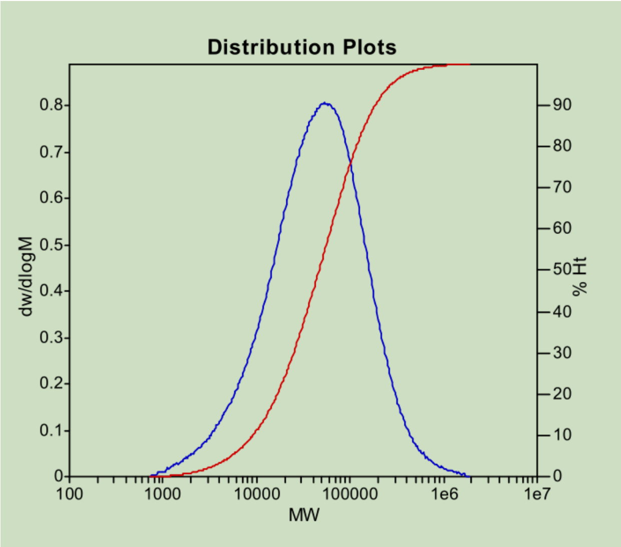

(A)

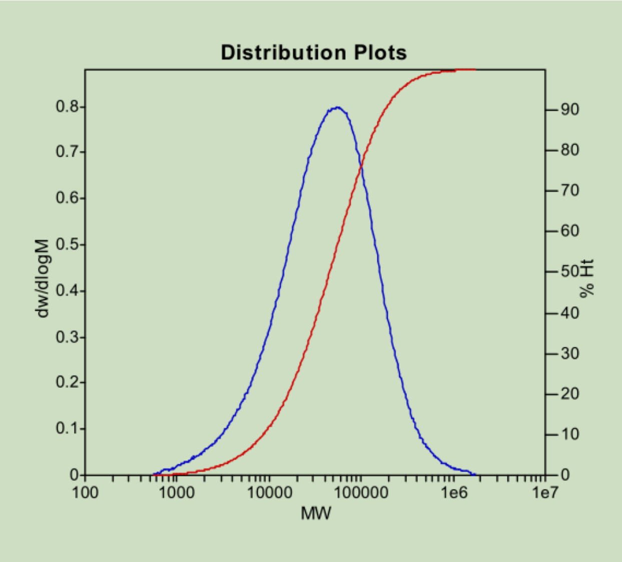

(B)

Figure S2. GPC analysis of the PE treated by the LCFBM medium incubated without (A) or with (B) strain Rd-H2 for 30 days.

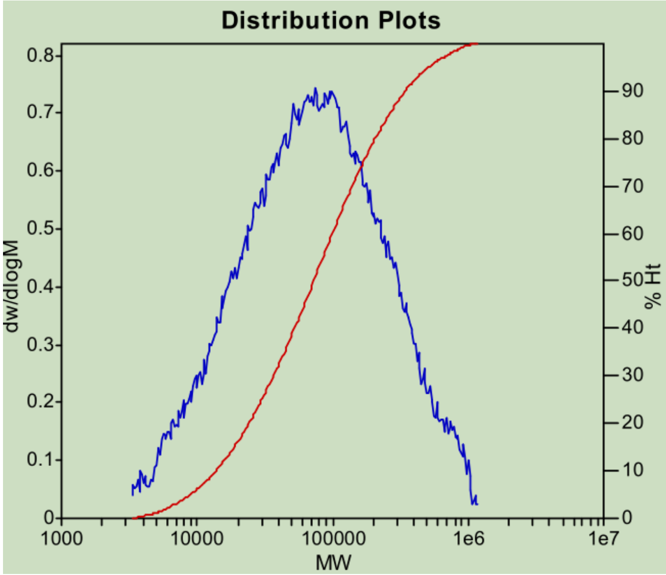

(A)

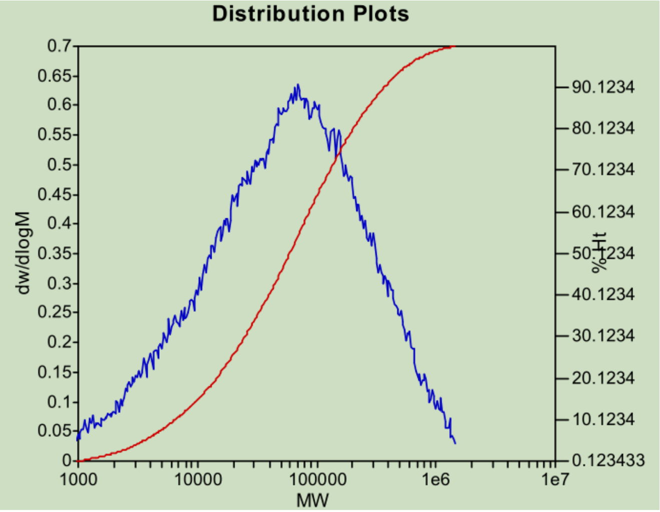

(B)

Figure S3. GPC analysis of the PE treated without (A) or with (B) AbMCO for 10h.

The sequence of the sequencing result of the pET-28a-AbMco:

GATCCGCAATTAAGGAATATCACCTTAATATTAATGAACAACAAGTCAATGTTACGGGCAAACCCCTTAAAC
GAATTACCGTAAATGGAAAATTTACAGCACCACTCCTTGAGTTTGAAGAGGGGGATGAGGCTGTTATTCGCG
TCCATAATCAATTAAAGAATCAAGATACCTCTTTACACTGGCATGGGTTGTTACTGCCAGGGTTAATGGATGG
TGTGCCGGGTTTTAATAATTTTAAAGGGATTGCTCCAAATGGAGATTTTGTTTACCGCTTTAAGGTGAAACAA
AATGGCACATATTGGTATCACGCCCATAGTAAAGGCCAAGAGCAAGACGGTTTATATGGGCCGCTTGTGATT
TACCCAAAAGGCAAAATACCAGTTGCGGCGCATGAAAAAACTGATCGTGATTATGTAGTGATGCTTTCAGAT
TTCCATGATTCTTCTAGCGATAGCATTATGAAGAATTTGAAAAAATCTGCTGAGTATTATCAAAACCGCCGTG
AAACTGTATCTGATGTATGGAAGCAAGTTAAAGCACAGGGCTTAAAAGCAACTTGGCAAGATCGTTCGATGT
GGAATCAGATGCGGATGTTAAAAACCGATATGTCTGATGTGACGGGCTATACCTTCTTAGTCAACGGCAAGA
CTCCTCAGCAAAACTGGACAGCTAATTTTAAAGCTGGTGATAAGGTTCGATTGCGATTTATTAATGCATCAGC
AATGTCATTTTTTGATGTCAGAATTCCAAATTTGAAAATGACAGTTGTAAGTGCAGATGGGCAGCCAGTTAAA

CCTGTAGCAATTGATGAGTTTCGTATTGGTACAGCTGAAACCTACGATGTCATTGTGGAACCTAAACAGCCAA
ATTATCAGATTGAAGCAGAGTCTATTGACCGTAGTGGTTTTGCTATCGGTACTTTGCACAATGAAAATACGCA
GGCTGTGGGGCCAGTCCAAATGCCAATACCACGCCCACGTGCTTTATTGACCATGGATGATATGGGCATGAG
TCATGGTGATGGAAGTAATGAACATGCTGGCCATCAAATGAACATGCAACATGACATGTCGGCCATGCCTGA
AATGAAAAAAGAGACGAGTCAGGCTAATCATGATCACACAGTGATGAAAATGGATCATGACATGAAAAATA
TGTCATCTGGCGAACATGATCATTCCATGATGCACATGAAGCATGATATGTCAGCGATGTCTGAAATGAAGC
ATGATATGCAGGCAATGTCATCTAGCGAGCATGACCACACTATGATGAATATGAATCATGAGATGCCTGCTC
AATCTGAGAATAAGTCTAAAAAGGATGAACCAGTTTATGGCTGGGCAAATGCTTCAACGCCAGCGGGTATGA
AAGCACTGCAATATAGCGATTTGCAATCTTTAACACCTCAGCCAGATACCCGTGCGCCCGAACGAGAATTAG
TCATTCGTTTAGGTGGCAACATGGAGCGTTATATCTGGACCATTAATGGCAAGAAATTTAGTGACACGATACC
TTTACAAGTTAAGTATGGTGAACGCATACGCTTGAAGTTTGTGAATGACAGTATGATGGCTCATCCAATGCAT
TTACATGGCATGTTTATGCAATTGGAAAATGGTCAACAAGCTGAAAATTTACCAAATAAACATACTATTATTG
TTCCACCCGGAAAGACCGTTACGGCGTTATTGACAGCCGATGCTTTGGGAGAATGGGCGATTCATTGCCATCT
GCTCTATCACATGAGTGCTGGAATGATGAATAAATTAATTGTTGCTCAAGTCAACGATGGACAACAACCCGC
TCAGTCCCAGCCAACTGTTCAATCTGAAAATGAGAAAGGAGCTAGTCATGCGCATCACTAA
